# Effects of blood flow restriction combined with visual feedback training on lower limb function and the serum levels of BDNF and VEGF in patients with stroke

**DOI:** 10.1371/journal.pone.0358740

**Published:** 2026-09-28

**Authors:** Hai Jiang, Chengzhuo Yang, Pan Wang, Mei Yu, Yijun Wang, Cuiting Wang, Deng Xiao

**Affiliations:** Department of Rehabilitation Medicine, The Affiliated Rehabilitation Hospital of Chongqing Medical University, Chongqing, China; TIU: Tishk International University, IRAQ

## Abstract

**Objective:**

This study aimed to investigate the effects of blood flow restriction training (BFRT) combined with visual feedback training (VFT) on lower limb function and serum levels of brain-derived neurotrophic factor (BDNF) and vascular endothelial growth factor (VEGF) in patients with stroke.

**Methods:**

Forty patients with stroke who were hospitalized at the Rehabilitation Hospital of Chongqing Medical University between July 2025 and May 2026 were enrolled and randomly assigned to either the VFT group (control group) or the BFRT combined with VFT group (experimental group). The control group received VFT in addition to conventional rehabilitation, whereas the experimental group received BFRT combined with VFT on the basis of conventional rehabilitation. After 4 weeks of treatment, manual muscle testing (MMT), the Fugl-Meyer Assessment-Lower Extremity (FMA-LE), the Timed Up and Go Test (TUGT), the Modified Barthel Index (MBI), the Functional Ambulation Category (FAC), the Pro-Kin balance test, and serum levels of BDNF and VEGF were compared between the two groups.

**Results:**

Compared with baseline, MMT, FMA-LE, MBI, and FAC scores significantly increased in both groups after 4 weeks of treatment (*P* < 0.05), with greater improvements observed in the experimental group than in the control group (*P* < 0.05). TUGT performance also improved significantly in both groups compared with baseline (*P* < 0.05), with the experimental group demonstrating significantly greater improvement than the control group (*P* < 0.05). Following treatment, Pro-Kin balance test performance improved significantly in both groups (*P* < 0.05), and the experimental group achieved better results than the control group (*P* < 0.05). Serum levels of BDNF and VEGF increased significantly in both groups after the intervention (*P* < 0.05), with significantly greater increases observed in the experimental group (*P* < 0.05).

**Conclusion:**

BFRT combined with VFT can significantly improve balance, lower limb function, and activities of daily living in patients with stroke. It also effectively increases serum levels of BDNF and VEGF, thereby promoting neurovascular plasticity.

## 1 Introduction

Stroke is a severe neurological disease and is the second leading cause of death and the third leading cause of disability worldwide. It is characterized by high incidence, disability, mortality, and recurrence rates [1]. More than 2 million new cases of stroke are reported annually in China [2], and approximately 55%−75% of patients experience long-term limb motor dysfunction [3]. Lower limb motor dysfunction is one of the most common impairments following stroke. Muscle weakness on the hemiplegic side, together with abnormalities in muscle tone and proprioception, impairs balance and walking ability, increases the risk of falls, and seriously affects activities of daily living [4,5]. Exercise-based rehabilitation and functional reconstruction play essential roles in stroke rehabilitation [6]. However, improving balance and walking ability in patients with stroke remains a major challenge that urgently needs to be addressed in the field of rehabilitation medicine [7].

The strength of the lower limb muscle groups plays a crucial role in balance and walking ability in patients with stroke. High-intensity exercise is the foundation of muscle strength and endurance training. However, patients with stroke are often unable to perform high-intensity resistance training because of prolonged bed rest or reduced exercise tolerance [8,9]. Blood flow restriction training (BFRT) can achieve effects comparable to those of high-load training while using low-load exercise, and its safety and efficacy have been well established [10,11] BFRT is an emerging rehabilitation intervention that applies external pressure to the proximal upper or lower limbs using a BFRT device. By partially restricting arterial inflow and venous outflow, BFRT increases muscle tension and places the muscles in a simulated high-intensity exercise state [12]. BFRT may promote muscle hypertrophy and increase muscle strength by enhancing muscle metabolism and stimulating growth hormone secretion [13,14]. It has been applied in neurorehabilitation through approaches such as low-intensity resistance training [15] and intermittent BFRT [16] to enhance muscle strength in patients with stroke, thereby improving balance, walking ability, and activities of daily living.

Studies have shown that a brain-limb synergistic regulatory approach can more effectively improve lower limb motor function in patients with stroke [17]. This approach integrates evidence-based interventions targeting both the brain and the limbs, thereby synergistically enhancing brain function, facilitating neurological recovery, and optimizing therapeutic outcomes. Yun and colleagues [18] used mirror therapy combined with neuromuscular electrical stimulation to improve hand function in patients with stroke. Chu and co-workers [19] combined transcranial direct current stimulation with isometric strength training to improve lower limb motor function and walking ability in patients with chronic stroke. Another study reported that sit-to-stand training combined with real-time visual feedback effectively improved muscle strength, balance, and walking ability in patients with stroke [20]. Visual feedback training (VFT) has been shown to improve balance and lower limb function by enhancing movement awareness, correcting abnormal movement patterns, and improving movement accuracy and motor control [21]. These effects may be mediated through activation of the cerebral sensorimotor cortex and reinforcement of motor learning, thereby promoting functional reorganization of the central nervous system [22]. In summary, VFT promotes functional reorganization of the central nervous system, whereas BFRT enhances peripheral muscle adaptation and local metabolic responses. Through a brain-limb synergistic regulatory approach that combines central motor learning with peripheral physiological adaptation, the combination of BFRT and VFT may improve balance and walking ability in patients with stroke more effectively than VFT alone. Therefore, this study aimed to compare the effects of VFT alone with those of BFRT combined with VFT on lower limb function, balance, walking ability, activities of daily living, and serum levels of BDNF and VEGF in patients with stroke. Furthermore, this study explored the potential mechanisms underlying these effects, thereby providing a theoretical basis for the rehabilitation of balance and walking dysfunction after stroke.

## 1 Materials and methods

### 1.1 General information

This study was approved by the Ethics Committee of the Rehabilitation Hospital of Chongqing Medical University (Approval No. 2025 Ethics Approval No. 10) and registered with the Chinese Clinical Trial Registry (Registration No. ChiCTR2500104181). Before enrollment, all participants were screened according to the predefined eligibility criteria. They were required to have stable vital signs, clear consciousness, sufficient cognitive and communicative capacity to understand and cooperate with the study procedures, and good compliance. Written informed consent was obtained from all participants or their legal guardians before enrollment.

A total of 52 patients with stroke who were hospitalized at the Rehabilitation Hospital of Chongqing Medical University between July 2025 and May 2026 were assessed for eligibility. The inclusion criteria were as follows: (1) diagnosis of stroke according to the diagnostic criteria established at the Fourth National Academic Conference on Cerebrovascular Diseases and confirmed by cranial CT or MRI; (2) within 6 months after the first-ever stroke; (3) aged 40–70 years; (4) stable vital signs, clear consciousness, good compliance, and the ability to understand and cooperate with the study procedures; (5) Holden Functional Ambulation Classification grade ≥2; (6) adequate cardiopulmonary function and the ability to tolerate rehabilitation training; and (7) provision of written informed consent. Both patients with ischemic stroke and those with hemorrhagic stroke were eligible because the intervention targeted common post-stroke impairments in lower limb motor function, balance, walking ability, and activities of daily living. Eligibility was determined on the basis of clinical diagnosis, neuroimaging findings, and the predefined functional and safety criteria.

The exclusion criteria were as follows: (1) bone or joint diseases, such as a history of fracture surgery or osteoarthritis; (2) malignant hypertension; (3) malignant tumors; (4) thrombosis; and (5) a history of psychiatric disorders.

The termination and dropout criteria were as follows: (1) voluntary withdrawal by the participant or their family member; (2) the investigator’s determination that continued participation might pose a risk to the participant; (3) occurrence of serious complications or other diseases during the study; (4) poor tolerance preventing completion of the intervention; or (5) inability to complete the study for other reasons.

A priori sample-size estimation was performed using G*Power version 3.1. The calculation was based on an analysis of variance for repeated measures examining the group × time interaction, with the Pro-Kin balance test and training system assessment designated as the primary outcome. Assuming a moderate effect size of f = 0.25, a two-sided significance level of α = 0.05, and a statistical power of 80%, the estimated minimum sample size was 34 participants. Allowing for an anticipated dropout rate of approximately 10%−15%, the target sample size was set at 40 participants.

Of the 52 patients assessed for eligibility, 9 did not meet the inclusion criteria and 3 declined to participate. The remaining 40 eligible patients provided written informed consent and were enrolled in the study. Participants were then randomly assigned identification numbers from 1 to 40 and allocated to the control group (n = 20) or experimental group (n = 20) using a random-number table. The study flowchart is presented in Fig 1.

**Fig 1:**
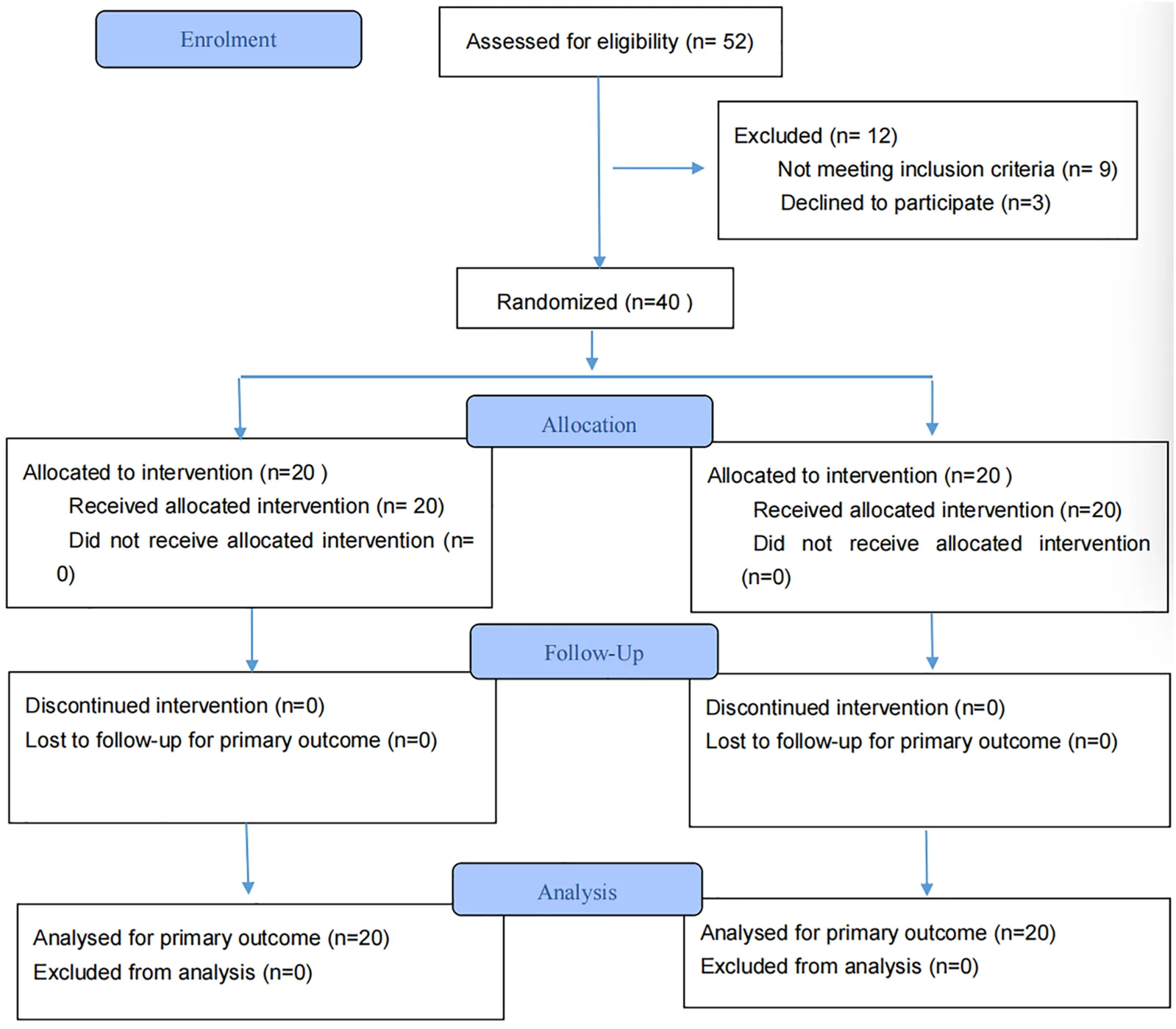
Flowchart of the study.

### 1.2 Research methods

The primary outcomes were balance and gait-related function, assessed before treatment and after 4 weeks of intervention. Balance was evaluated using the Pro-Kin balance test and training system, including the limit of stability, movement trajectory area, and movement trajectory length under eyes-open and eyes-closed conditions. Secondary outcomes included lower limb muscle strength assessed by manual muscle testing, lower limb motor function assessed using the Fugl-Meyer Assessment of the Lower Extremity (FMA-LE), mobility assessed using the Timed Up and Go Test (TUGT), walking ability assessed using the Functional Ambulation Category (FAC), activities of daily living assessed using the Modified Barthel Index (MBI), and serum levels of brain-derived neurotrophic factor (BDNF) and vascular endothelial growth factor (VEGF).

Both groups received conventional rehabilitation training. In addition, the control group received VFT, whereas the experimental group received a combination of VFT and BFRT. Both VFT and BFRT interventions were supervised by professionally trained physical therapists.

#### 1.2.1 Conventional rehabilitation training.

Conventional rehabilitation training included physical therapy (core stability training, balance and coordination training, walking training, etc.), occupational therapy, physical factor therapy (electrical stimulation, thermotherapy, etc.), and traditional rehabilitation training. Physical therapy and occupational therapy were each performed for 30 min/session/day, 5 days/week. Physical factor therapy and traditional rehabilitation training were each performed for 20 min/session/day. All interventions were conducted 5 days per week for a total duration of 4 weeks.

#### 1.2.2 VFT.

VFT was performed using the PK-252 Pro-Kin visual feedback balance training system (TECNOBODY, Italy). Based on the patients’ functional status and personal interests, therapists selected appropriate game-based training programs, including table tennis, balance ball, skiing, and fruit slicing games. During training, patients stood on the balance training platform and controlled the movement of a cursor on the game interface by maintaining and shifting their center of gravity to complete the task-oriented exercises. Training was performed for 20 min/session/day, 5 days/week, for a total of 4 weeks.

#### 1.2.3 BFRT.

During VFT, BFRT was performed using a pressurized BFRT cuff (Theratools, Henan Heju Rehabilitation Technology Co., Ltd.; cuff width, 10 cm; cuff length, 109 cm). The cuff was placed around the proximal thigh of the affected limb, as close to the trunk as possible. The inflation tube of the air pump was connected to the cuff valve to enable pressure monitoring and regulation. The cuff pressure was maintained at 160–200 mmHg throughout the 20-minute VFT session. The fixed pressure range was selected based on the BFRT protocol used in our rehabilitation setting and previous studies reporting vascular occlusion pressures in a similar range during resistance exercise and stroke rehabilitation [15].

Arterial limb occlusion pressure (LOP) was not directly measured; therefore, the pressure was not prescribed as a percentage of individual LOP. No additional resistance exercise was performed under BFRT, and the BFRT exposure consisted of 20 min/session, 5 days/week for 4 weeks, concurrently with VFT. During training, blood pressure, oxygen saturation, and cuff pressure were monitored every 5 min. Participants were also continuously monitored for numbness, pain, fatigue, changes in limb circumference, skin color, and other signs of intolerance. Training was immediately adjusted or discontinued if clinically concerning symptoms or abnormal changes occurred. The duration of BFRT was the same as that of VFT in the experimental group.

### 1.3 Assessment tools

Before treatment and after 4 weeks of intervention, all patients were assessed by designated evaluators who were blinded to group allocation.

#### 1.3.1 Assessment of muscle strength.

Muscle strength was assessed using the Lovett manual muscle testing system, which consists of six grades (0, 1, 2, 3, 4, and 5). Higher scores indicate greater muscle strength [23].

#### 1.3.2 FMA-LE.

The FMA-LE consists of 17 items, including reflex activity and synergistic movement. Each item is scored on a 3-point scale (0–2), with a maximum total score of 34 points. Higher scores indicate better lower limb motor function [24].

#### 1.3.3 TUGT.

A marker was placed 3 m in front of a chair with a backrest. The patient started from a seated position with their back against the chair. Timing began when the patient’s buttocks lifted from the chair. After standing, the patient walked at a comfortable speed to the marker, turned around, returned to the chair, and sat down in the original position. The total time required to complete the task was recorded in seconds (s). The test was repeated twice, with a 1-minute rest interval between trials, and the average value was used for analysis [25].

#### 1.3.4 FAC.

The FAC consists of six grades (0–5), with higher scores indicating better walking ability [26].

#### 1.3.5 MBI.

The MBI consists of 10 items, including bathing and grooming, with a maximum total score of 100 points. Scores are assigned according to the level of assistance required to complete daily activities. Higher scores indicate better functional independence [27].

#### 1.3.6 Pro-Kin balance test.

The limit of stability (LoS) represents the maximum angle that an individual can achieve by voluntarily leaning while maintaining a standing position. A higher LoS value indicates better balance ability and a lower risk of falls. The length of the movement trajectory represents the total distance traveled by the center of pressure (COP), with shorter trajectories indicating better balance performance. The movement trajectory area refers to the area enclosed by the COP trajectory, with smaller areas indicating better balance function [28].

#### 1.3.7 Detection of serum biomarkers.

Peripheral venous blood samples (5 mL) were collected immediately after the final training session. Blood samples were allowed to stand at room temperature for 60 min, and were then centrifuged at 2,000 r·min^-1^ for 20 min. The resulting serum was separated and stored at −80°C until analysis. Serum BDNF and VEGF concentrations were determined using enzyme-linked immunosorbent assay (ELISA) kits according to the manufacturers’ instructions [29]. The BDNF ELISA kit was obtained from RUPIN BIOLOGY (catalog no. RP-12065). The assay range was 1.5625–47.86 ng/mL. The intra-assay coefficients of variation (CVs) were 4.63% and 3.38% for Levels 1 and 2, respectively, and the inter-assay CVs were 10.32% and 8.85%, respectively. These values were within the manufacturer-reported acceptable limits (<10% for intra-assay precision and <15% for inter-assay precision).

### 1.4 Quality control

In this study, a rehabilitation therapist who participated in the trial was assigned to assist with research data collection. To ensure the objectivity and reliability of the collected data, the assessor was blinded to patient grouping, treatment allocation, and other relevant clinical information during the data collection process.

### 1.5 Statistical analysis

Statistical analyses were performed using SPSS version 27. Categorical data are presented as n (%), and comparisons were performed using the χ^2^ test. Normally distributed continuous variables are expressed as mean ± standard deviation (x̄ ± s), whereas non-normally distributed continuous variables are presented as median (interquartile range). For continuous variables with normal distribution and homogeneity of variance, comparisons between two groups were performed using the independent-samples t-test; otherwise, the Mann-Whitney U test was applied. Ordinal data between groups were compared using the Mann-Whitney U test, while within-group comparisons before and after intervention were performed using the paired Wilcoxon signed-rank test. A *P* value < 0.05 was considered statistically significant.

## 2 Results

### 2.1 Participant characteristics and study completion

A total of 40 participants were randomized, and all 40 completed the 4-week intervention and follow-up assessments. No adverse events, serious adverse events, protocol deviations requiring withdrawal, or treatment-related complications were observed during the intervention period. The baseline characteristics of the two groups are presented in Table 1. There were no significant between-group differences in sex distribution, age, disease duration, or stroke type (all *P* > 0.05), indicating that the two groups were comparable at baseline.

**Table 1:** Baseline characteristics of the two groups.

Variable	Control group (n = 20)	Experimental group (n = 20)	Test statistic	*P* value
Sex, n (%)			χ^2^ = 0.440	0.507
Male	12 (60.0)	14 (70.0)		
Female	8 (40.0)	6 (30.0)		
Age, years	58.05 ± 8.13	56.35 ± 8.71	*t* = 0.638	0.527
Disease duration, days	38.90 ± 9.41	39.20 ± 11.84	*t* = 0.089	0.930
Stroke type, n (%)			χ^2^ = 0.102	0.749
Ischemic stroke	11 (55.0)	12 (60.0)		
Hemorrhagic stroke	9 (45.0)	8 (40.0)		

Data are presented as n (%) or mean ± SD. Categorical variables were compared using the χ^2^ test, and continuous variables were compared using the independent-samples *t*-test.

### 2.2 Comparison of lower limb muscle strength on the hemiplegic side before and after treatment

At baseline, no significant between-group differences were observed in the assessed lower limb muscle-strength measures (Table 2). After 4 weeks of intervention, muscle strength improved in both groups. The experimental group showed greater improvements in hip flexion, hip extension, hip abduction, knee flexion, knee extension, ankle dorsiflexion, and ankle plantar flexion than the control group. In ordinal regression analyses, the adjusted odds ratios for the between-group effects ranged from 0.050 to 0.230, with corresponding 95% CIs of 0.008–0.938 and *P* values ranging from 0.001 to 0.040 (Table 2). The complete baseline and post-treatment distributions and effect estimates are provided in Fig 2 and Table 4.

**Table 2:** Numerical results for ordinal lower-limb muscle-strength and functional outcomes before and after the 4-week intervention.

Outcome	Control baseline, median (IQR)	Control post, median (IQR)	Experimental baseline, median (IQR)	Experimental post, median (IQR)	Effect estimate (aOR)	95% CI	*P*
Hip flexion MMT	3 (2, 3)	3 (3, 4)	2.5 (2, 3)	4 (3.25, 4)	0.061	0.011–0.352	0.002
Hip extension MMT	2 (1, 2)	2.5 (2, 3)	2 (1, 2)	3 (3, 3)	0.050	0.008–0.316	0.001
Hip abduction MMT	2 (2, 2)	3 (3, 3)	2 (2, 2.75)	3.5 (3, 4)	0.202	0.051–0.801	0.023
Knee flexion MMT	3 (2, 3)	3 (3, 4)	3 (2, 3)	4 (4, 4)	0.194	0.051–0.736	0.016
Knee extension MMT	2 (1, 2)	3 (2, 3)	2 (1, 2)	3 (3, 4)	0.066	0.011–0.390	0.003
Ankle dorsiflexion MMT	2 (1, 2)	3 (2, 3)	2 (1, 2)	3 (3, 3.75)	0.230	0.056–0.938	0.040
Ankle plantar flexion MMT	2 (2, 2)	3 (2, 3)	2 (2, 2.75)	3 (3, 4)	0.081	0.013–0.511	0.008
FAC	2 (2, 3)	3 (3, 4)	2 (2, 3)	4 (3.25, 4)	0.148	0.037–0.587	0.007

**Fig 2:**
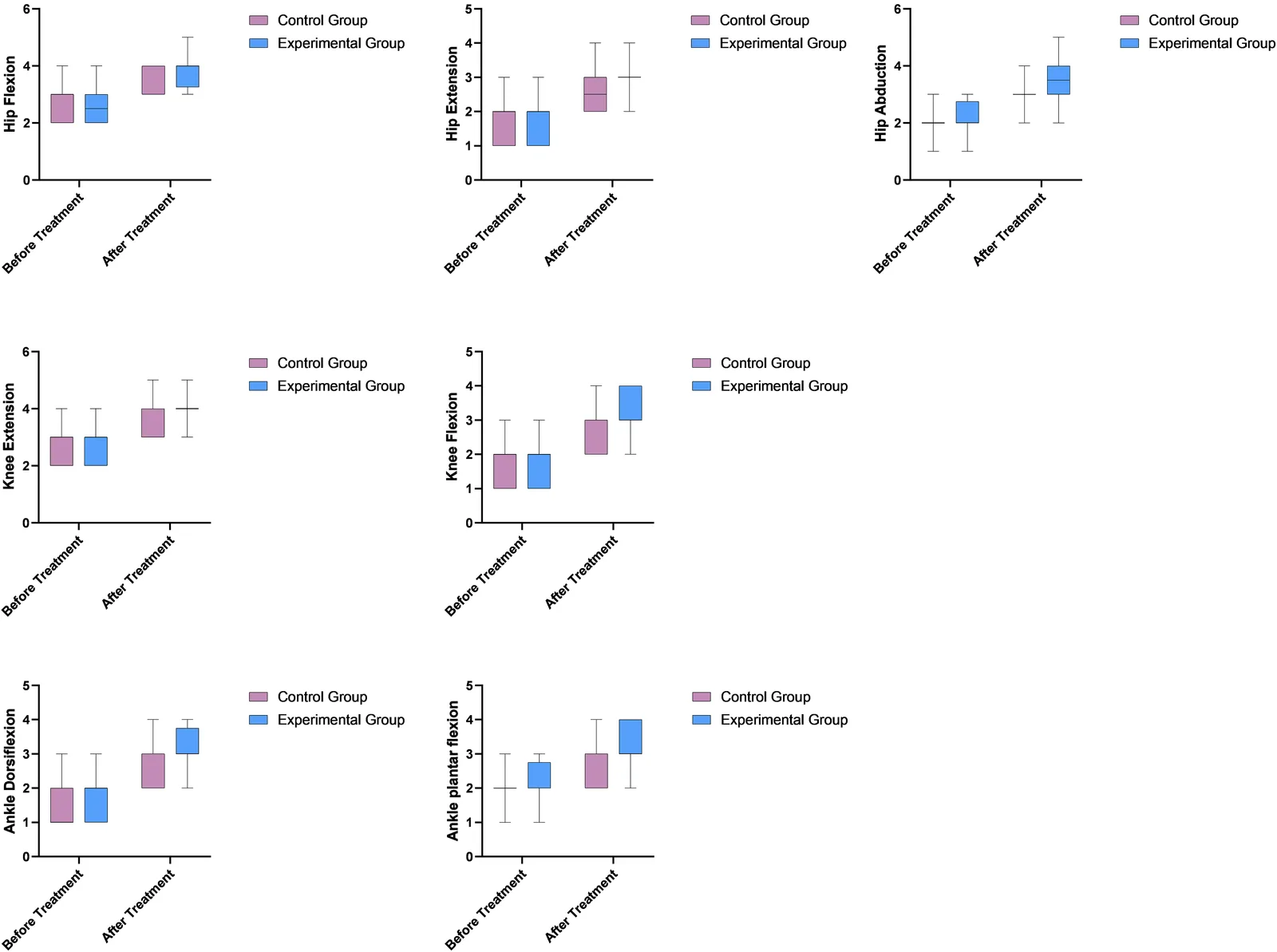
Box plot showing between-group comparisons of lower limb muscle strength before and after treatment, including hip flexion, hip extension, hip abduction, knee extension, knee flexion, ankle dorsiflexion, and ankle plantar flexion.

### 2.3 Between-group comparison of FMA-LE, TUGT, FAC, and MBI scores before and after treatment

There were no significant between-group differences in FMA-LE, TUGT, FAC, or MBI scores at baseline (Table 3). After 4 weeks, FMA-LE increased from 12.10 ± 3.09 to 19.85 ± 2.76 in the control group and from 12.05 ± 3.03 to 22.20 ± 1.96 in the experimental group. The adjusted between-group difference was −2.371 (95% CI, −3.681 to −1.061; *P* < 0.001; adjusted *P* < 0.001), favoring the experimental intervention.

**Table 3:** Numerical results for secondary and exploratory continuous outcomes before and after the 4-week intervention.

Outcome	Control (baseline)	Control (post)	Experimental (baseline)	Experimental (post)	Adjusted difference	95% CI	*P*	Adjusted *P*
FMA-LE	12.10 ± 3.09	19.85 ± 2.76	12.05 ± 3.03	22.20 ± 1.96	−2.371	−3.681 to −1.061	<0.001	<0.001
TUGT	26.40 ± 5.84	19.15 ± 3.80	25.70 ± 4.52	15.40 ± 4.44	7.817	4.441 to 11.194	<0.001	<0.001
MBI	50.35 ± 10.90	70.65 ± 11.53	50.65 ± 9.78	80.25 ± 8.25	−9.454	−15.084 to −3.824	0.002	0.010
BDNF	8.63 ± 1.61	13.99 ± 2.87	8.66 ± 1.80	16.63 ± 3.38	−2.629	−4.502 to −0.757	0.007	0.035
VEGF	89.03 ± 8.12	105.06 ± 8.03	86.73 ± 8.29	112.21 ± 8.10	−7.479	−12.714 to −2.244	0.006	0.030

TUGT completion time decreased from 26.40 ± 5.84 to 19.15 ± 3.80 s in the control group and from 25.70 ± 4.52 to 15.40 ± 4.44 s in the experimental group. The adjusted between-group difference was 7.817 (95% CI, 4.441–11.194; *P* < 0.001; adjusted *P* < 0.001).

MBI increased from 50.35 ± 10.90 to 70.65 ± 11.53 in the control group and from 50.65 ± 9.78 to 80.25 ± 8.25 in the experimental group. The adjusted between-group difference was −9.454 (95% CI, −15.084 to −3.824; *P* = 0.002; adjusted *P* = 0.010). All results are summarized in Table 3 and Fig 3.

**Fig 3:**
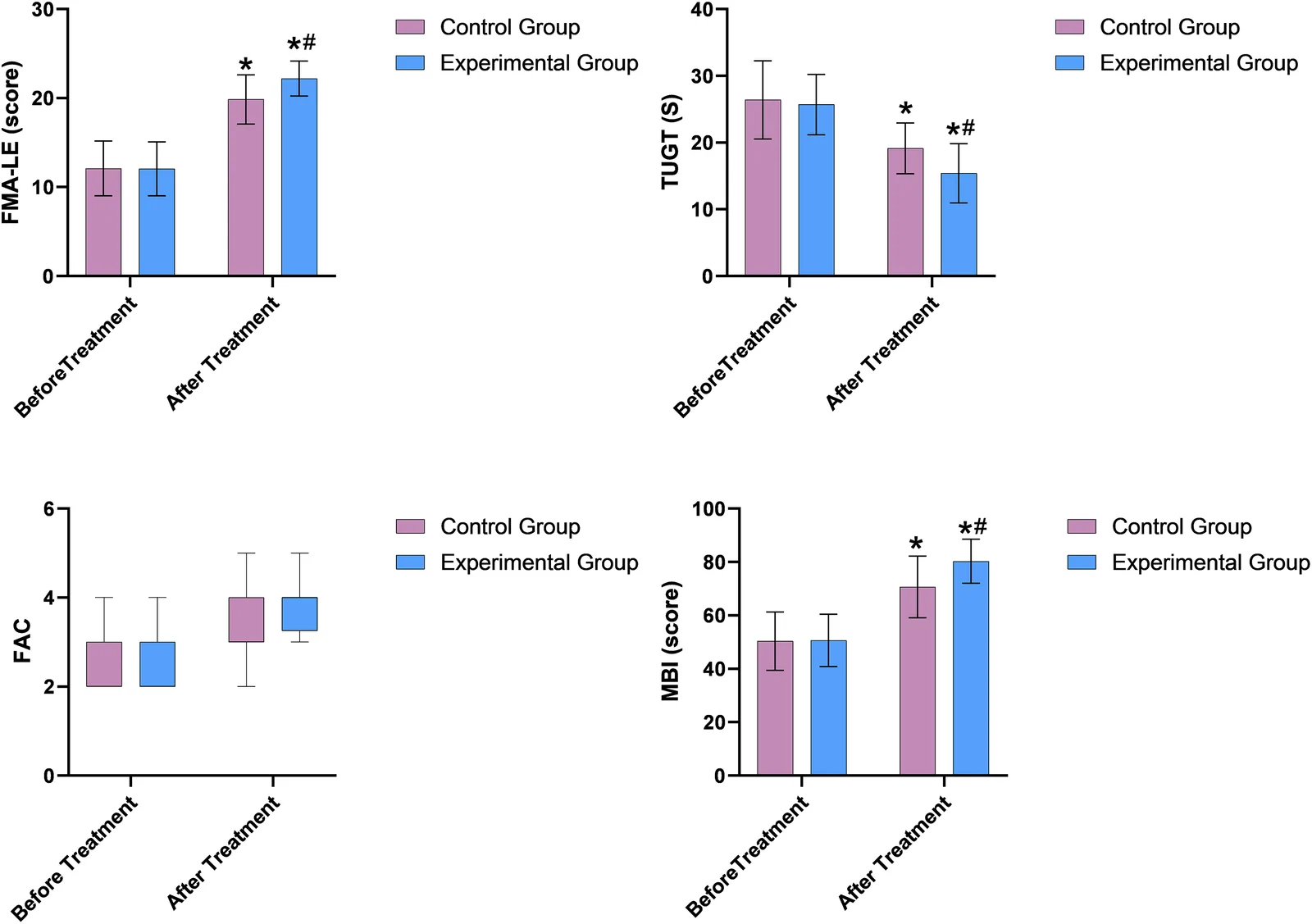
Between-group comparisons of FMA-LE, TUGT, FAC, and MBI scores before and after treatment. ^*^P < 0.05 compared with before treatment; ^#^*P* < 0.05 compared with the control group.

### 2.4 Between-group comparison of Pro-Kin balance test results before and after treatment

No significant baseline differences were observed between groups for the Pro-Kin limit of stability, trajectory area, or trajectory length under either eyes-open or eyes-closed conditions (Table 4).

**Table 4:** Numerical results for the primary Pro-Kin balance outcomes before and after the 4-week intervention.

Primary outcome	Control (baseline)	Control (post)	Experimental (baseline)	Experimental (post)	Adjusted between-group difference	95% CI	P
Pro-Kin limit of stability	31.46 ± 9.25	56.56 ± 12.41	34.57 ± 11.06	73.67 ± 10.62	−15.65	−22.54 to −8.75	<0.001
Pro-Kin trajectory area, eyes open	382.05 ± 134.41	328.60 ± 85.49	403.40 ± 58.18	281.35 ± 51.02	57.54	25.12 to 89.96	<0.001
Pro-Kin trajectory area, eyes closed	569.95 ± 151.73	492.30 ± 200.11	572.15 ± 110.77	390.40 ± 42.80	103.30	27.12 to 179.49	0.009
Pro-Kin trajectory length, eyes open	507.75 ± 88.07	436.90 ± 101.92	517.45 ± 68.32	373.35 ± 63.72	70.30	28.03 to 112.56	0.002
Pro-Kin trajectory length, eyes closed	716.20 ± 84.54	581.35 ± 175.75	708.20 ± 76.45	485.20 ± 68.88	93.04	8.68 to 177.39	0.032

After 4 weeks of intervention, the limit of stability increased from 31.46 ± 9.25 to 56.56 ± 12.41 in the control group and from 34.57 ± 11.06 to 73.67 ± 10.62 in the experimental group. The adjusted between-group difference was −15.65 (95% CI, −22.54 to −8.75; *P* < 0.001).

For trajectory area with eyes open, values decreased from 382.05 ± 134.41 to 328.60 ± 85.49 in the control group and from 403.40 ± 58.18 to 281.35 ± 51.02 in the experimental group, corresponding to an adjusted between-group difference of 57.54 (95% CI, 25.12–89.96; *P* < 0.001). Under eyes-closed conditions, trajectory area decreased from 569.95 ± 151.73 to 492.30 ± 200.11 in the control group and from 572.15 ± 110.77 to 390.40 ± 42.80 in the experimental group, with an adjusted between-group difference of 103.30 (95% CI, 27.12–179.49; *P* = 0.009).

Similarly, trajectory length under eyes-open conditions decreased from 507.75 ± 88.07 to 436.90 ± 101.92 in the control group and from 517.45 ± 68.32 to 373.35 ± 63.72 in the experimental group, with an adjusted between-group difference of 70.30 (95% CI, 28.03–112.56; *P* = 0.002). Under eyes-closed conditions, trajectory length decreased from 716.20 ± 84.54 to 581.35 ± 175.75 in the control group and from 708.20 ± 76.45 to 485.20 ± 68.88 in the experimental group, with an adjusted between-group difference of 93.04 (95% CI, 8.68–177.39; *P* = 0.032). These findings are summarized in Fig 4 and Table 4.

**Fig 4:**
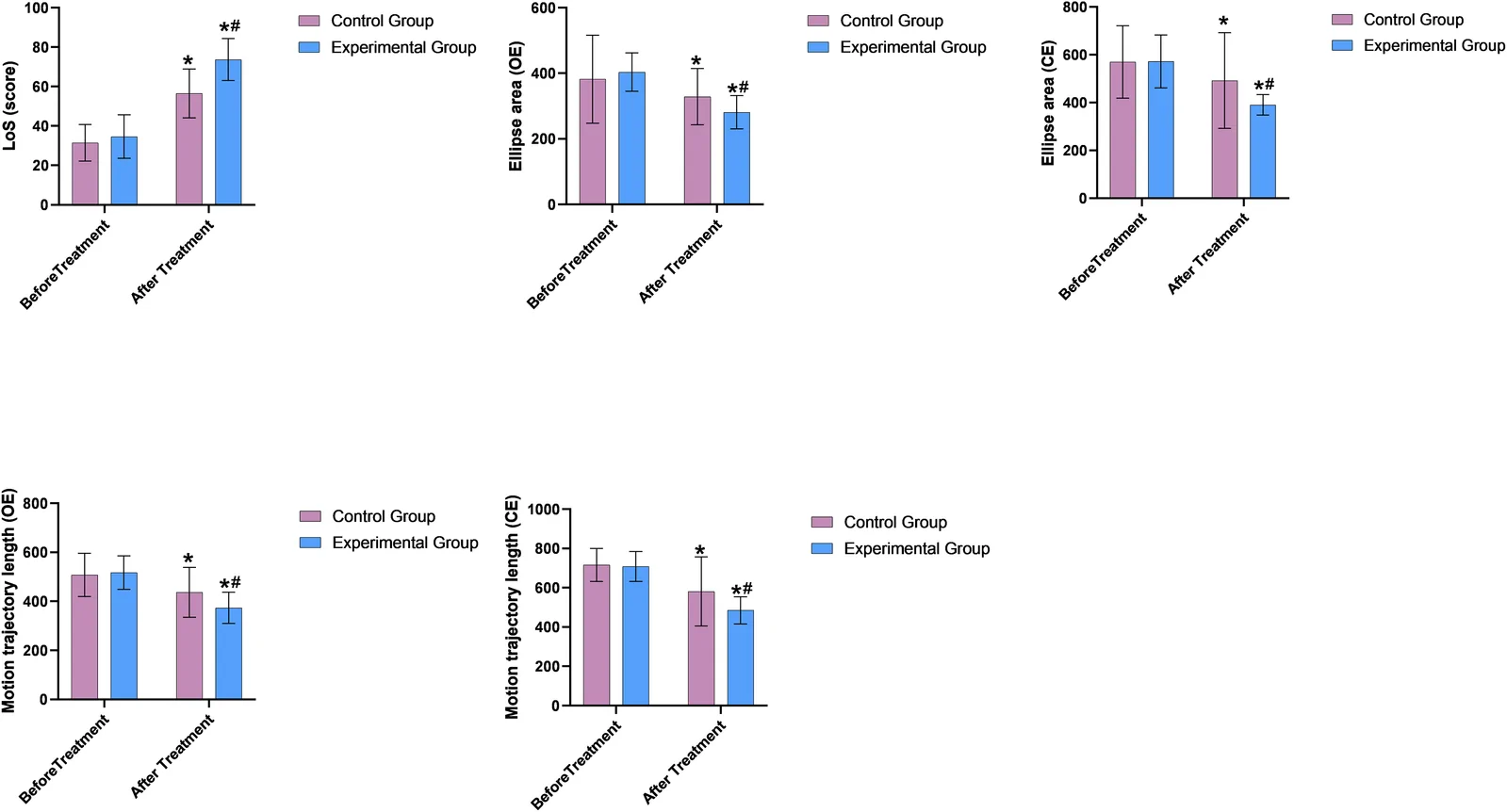
Between-group comparisons of Pro-Kin balance test parameters before and after treatment, including limit of stability, movement trajectory area (eyes open and eyes closed), and movement trajectory length (eyes open and eyes closed). ^*^P < 0.05 compared with before treatment; ^#^*P* < 0.05 compared with the control group.

### 2.5 Between-group comparison of serum BDNF and VEGF levels before and after treatment

Baseline serum BDNF and VEGF concentrations were comparable between the two groups (Table 3). After 4 weeks of intervention, serum BDNF increased from 8.63 ± 1.61 to 13.99 ± 2.87 ng/mL in the control group and from 8.66 ± 1.80 to 16.63 ± 3.38 ng/mL in the experimental group. The adjusted between-group difference was −2.629 (95% CI, −4.502 to −0.757; P = 0.007; adjusted *P* = 0.035).

Serum VEGF increased from 89.03 ± 8.12 to 105.06 ± 8.03 ng/mL in the control group and from 86.73 ± 8.29 to 112.21 ± 8.10 ng/mL in the experimental group. The adjusted between-group difference was −7.479 (95% CI, −12.714 to −2.244; *P* = 0.006; adjusted *P* = 0.030). Complete results for BDNF and VEGF are presented in Fig 5 and Table 3.

**Fig 5:**
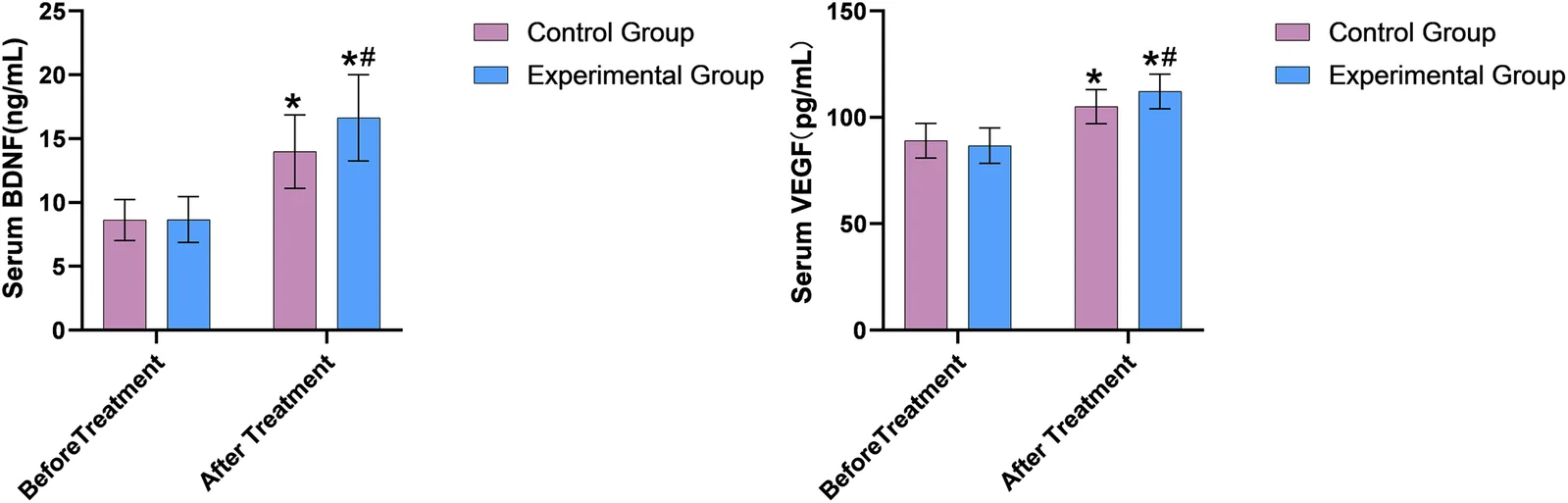
Between-group comparisons of serum BDNF and VEGF levels before and after treatment. ^*^P < 0.05 compared with before treatment; ^#^*P* < 0.05 compared with the control group.

## 3 Discussion

Stroke is a neurological disease with a high global disability burden and frequently results in severe lower limb motor dysfunction. It impairs balance control and walking ability, substantially increases the risk of falls, and imposes a significant socioeconomic burden [30]. Although conventional rehabilitation training remains the cornerstone of functional recovery, single-modality interventions have limited effects on promoting neuroplasticity and peripheral muscle adaptation, making it challenging to meet patients’ urgent need for effective motor function recovery [31]. Increasing evidence suggests that multimodal intervention strategies integrating central nervous system regulation with peripheral muscle strengthening may overcome these limitations. However, effectively coordinating different mechanisms to maximize rehabilitation outcomes remains a key challenge in current clinical research [32]. By combining BFRT with VFT, this study explored a rehabilitation strategy aimed at improving balance and walking ability in patients with stroke through a “peripheral-central” synergistic mechanism.

The results of this study demonstrated that after 4 weeks of intervention, BFRT combined with VFT effectively improved lower limb muscle strength, motor function, balance ability, walking ability, and activities of daily living in patients with post-stroke hemiplegia. Notably, the combined intervention produced greater benefits than VFT alone. Compared with VFT alone, which primarily relies on central nervous system remodeling, the combination of BFRT and VFT may more effectively facilitate the reconstruction of motor control strategies through a dual pathway involving “peripheral metabolic stress and central sensory input,” thereby overcoming the limitations of single interventions [33].

Whyte and colleagues [34] reported that the affected limb after stroke exhibits a specific pathophysiological state characterized by reduced muscle oxygen consumption and impaired microvascular reactivity. These alterations may limit the capacity of traditional low-load training to induce sufficient muscle adaptation. In the present study, the combination of BFRT and VFT significantly improved muscle strength in patients with post-stroke hemiplegia. BFRT performed concurrently with VFT may have induced a localized ischemic and hypoxic environment within skeletal muscle, promoting metabolite accumulation and generating metabolic stress similar to that observed during high-intensity exercise [35]. This metabolic stress may counteract impaired oxygen metabolism and microvascular dysfunction in the hemiplegic limbs and compensate for insufficient voluntary contraction capacity of the affected muscles. Furthermore, metabolic stress may activate signaling pathways, such as hypoxia-inducible factor pathways, thereby enhancing peripheral muscle stimulation beyond that achieved through purely central training approaches [11].

Several studies have reported that BFRT can effectively improve muscle strength in patients with stroke [36–39], which is consistent with the findings of the present study. By enhancing muscle strength, BFRT may facilitate proprioceptive recovery, improve joint stability, and promote selective activation of movements involving the hip, knee, and ankle on the hemiplegic side [40]. Moreover, BFRT not only increases the strength of blood flow-restricted muscle groups but may also enhance the performance of non-restricted muscle groups and improve trunk control in patients with hemiplegia, thereby contributing to improvements in balance and walking ability [41]. Although muscle strengthening is essential for optimizing overall motor function, improvements in muscle strength alone are insufficient to restore complex walking ability, highlighting the continued importance of central nervous system involvement.

The brain must integrate multiple sensory inputs, including visual, vestibular, and somatosensory information, to effectively regulate postural control and movement execution [42,43]. Patients with stroke exhibit delayed cortical activation during balance recovery, particularly when relying on the affected limb. This impairment in central processing speed may limit the recovery of dynamic balance [44]. VFT provides real-time feedback regarding movement errors and requires patients to engage greater cognitive resources to adjust their movements, thereby promoting rapid recruitment of the sensorimotor cortex [45]. Previous studies have demonstrated that VFT can improve balance, walking function, and activities of daily living in patients with stroke [46], which is consistent with the results of this study. In summary, BFRT may optimize the peripheral metabolic environment and enhance sensory input, thereby improving muscle activation efficiency and providing more accurate proprioceptive information. Meanwhile, VFT strengthens the ability of the central nervous system to receive and integrate sensory feedback and to develop appropriate motor strategies. Through a “peripheral-central” synergistic mechanism, BFRT and VFT may achieve comprehensive optimization from peripheral motor execution to central motor control. This synergistic interaction may underlie the superior effects of the combined intervention across multiple functional outcomes.

Serum levels of BDNF and VEGF are closely associated with functional recovery following brain injury, and functional remodeling of the brain represents a primary mechanism underlying limb functional recovery after stroke [47,48]. Therefore, this study measured serum BDNF and VEGF levels to further explore the potential mechanisms underlying the beneficial effects of BFRT combined with VFT in patients with stroke. BDNF is one of the most extensively expressed neurotrophic factors in the central nervous system and plays critical roles in synaptic plasticity, neuronal survival, and neurogenesis [49,50]. Increased BDNF expression after stroke may promote cortical functional reorganization around the lesion area and enhance compensatory neural pathways in the contralateral hemisphere [44]. VEGF primarily promotes the proliferation and migration of vascular endothelial cells, facilitates angiogenesis in the ischemic penumbra, improves local cerebral blood flow, and contributes to the establishment of a microenvironment conducive to neuronal repair [51]. The present study demonstrated that the combination of BFRT and VFT significantly increased serum levels of BDNF and VEGF in patients with stroke. These findings suggest that BFRT combined with VFT may promote neurovascular repair by enhancing BDNF- and VEGF-mediated processes, thereby contributing to improvements in limb motor function and balance. Zhang et al. [29] and Abdullahi et al. [52] further supported this hypothesis, reporting that functional recovery after stroke is often accompanied by enhanced BDNF-mediated neuroplasticity and VEGF-mediated vascular remodeling.

Du and co-workers [53] found that low-intensity BFRT effectively increased serum BDNF and VEGF levels after stroke. However, some studies involving patients with chronic stroke have reported no significant changes in BDNF following exercise interventions. This discrepancy may be related to differences in participant characteristics and intervention intensity. Specifically, the inclusion of patients in the subacute or early chronic stages of stroke and the higher metabolic demands induced by the combined intervention protocol in the present study may have increased the likelihood of activating neuroprotective responses [54]. In this study, patients received a combined intervention of BFRT and VFT. Compared with baseline, serum levels of BDNF and VEGF increased significantly in both groups after 4 weeks of intervention, with greater increases observed in the combination therapy group than in the control group. The peripheral metabolic stress induced by BFRT, including reduced oxygen availability and lactate accumulation, may activate intracellular signaling pathways and promote the expression and release of BDNF and VEGF. Furthermore, the combination of BFRT and VFT may exert synergistic effects through a brain-limb regulatory model that integrates central motor learning reinforcement with peripheral physiological adaptation. This interaction may enhance neuronal repair and functional reorganization, thereby improving lower limb function, balance, and activities of daily living in patients with stroke.

This study had several limitations. First, ultrasound-based assessment was not used to accurately quantify the degree of blood flow restriction during BFRT, which may have influenced the precision of intervention parameters and the comparability of outcomes. Second, the study had a relatively small sample size, employed a single-center design, and lacked long-term follow-up. Moreover, because ischemic and hemorrhagic stroke may differ in pathophysiology and recovery trajectory, stroke subtype heterogeneity may have introduced variability in treatment response. Potential selection bias during participant recruitment may have affected the generalizability and robustness of the findings. Third, neither the participants nor the therapists could be blinded to the BFRT intervention because of its nature, and no sham-cuff condition was included. Therefore, expectancy effects, attention effects, and other nonspecific effects associated with the additional BFRT intervention cannot be completely excluded. Future trials should consider incorporating a sham-BFRT condition and appropriate blinding procedures where feasible to better isolate the specific effects of blood flow restriction. In addition, this study evaluated only serum BDNF and VEGF levels and did not incorporate neuroimaging or electrophysiological assessments. Therefore, the underlying mechanisms of BFRT-induced functional recovery could not be fully elucidated. Future studies should validate these findings through large-scale, multicenter randomized controlled trials, further optimize BFRT parameters, and incorporate multimodal assessment approaches to clarify the mechanisms by which BFRT promotes brain functional reorganization and neurological recovery.

## 4 Conclusion

Compared with VFT alone, BFRT combined with VFT was associated with greater short-term improvements in lower limb muscle strength, motor function, balance, walking ability, and activities of daily living, accompanied by greater increases in circulating serum BDNF and VEGF concentrations after 4 weeks of intervention. These biomarker findings may indicate enhanced peripheral neurotrophic- and angiogenesis-related responses; however, they do not directly demonstrate cerebral neurovascular plasticity or neural repair. Further studies incorporating neuroimaging, electrophysiological, and other mechanistic assessments are needed to clarify whether these peripheral biomarker changes are associated with central neuroplastic adaptations and functional recovery.

## Supporting information

S1 File Trail protocal from Project Task Book. (PDF)

S2 File CONSORT_2025 editable checklist. (DOCX)
