# Supplementary material for: Effects of blood flow restriction combined with visual feedback training on lower limb function and the serum levels of BDNF and VEGF in patients with stroke
Source: PLoS One. 2026 Sep 28;21(9):e0358740. doi: 10.1371/journal.pone.0358740 (PMC13618907; doi:10.1371/journal.pone.0358740)
Supplement: Supplementary file 1 [file pone.0358740.s001.pdf]

二、Main body of the report

Research Work (General Objectives, Research Content and Acceptance Evaluation Indicators)

一、Overall Goals：

1.Publish 1-2 related papers；

2.Hold 1 internal continuing education session.

二、Research Content：

1.Research Content：

(1) To explore the effects and safety of blood flow restriction combined with visual feedback balance training on balance ability, lower limb motor function, walking function and activities of daily living in stroke patients by comparing the therapeutic outcomes between the experimental group (blood flow restriction + visual feedback balance training) and the control group (visual feedback balance training alone).

(2) To explore the clinical application of blood flow restriction therapy in neurological rehabilitation.

2.Research Objectives：

(1)To evaluate the efficacy of the combined training in functional improvement: To verify the rehabilitation effect of blood flow restriction combined with visual feedback balance training, so as to provide safe and effective diversified treatment for stroke patients.

(2)To promote clinical application: To popularize blood flow restriction therapy in neurological rehabilitation, explore its feasibility and effectiveness, and realize personalized and intelligent rehabilitation.

(3)To enhance patients' initiative: To increase patients' participation in rehabilitation, and further improve their self-management ability and treatment compliance.

3.Key Research Questions to Be Addressed:

(1) What are the rehabilitation effects of blood flow restriction combined with visual feedback balance training on balance ability, lower limb motor function, walking function and activities of daily living in stroke patients? And whether it is more effective than visual feedback balance training used alone?

(2) Does blood flow restriction training carry a risk of lower limb deep venous thrombosis?

4. Proposed Research Scheme and Feasibility Analysis (Including research methods, technical routes, experimental approaches and key technologies)

(1)Subject of study:

①Inclusion Criteria

1) Diagnosed with stroke in accordance with the criteria established at the 4th National Academic Conference on Cerebrovascular Diseases, and confirmed by cranial CT or MRI.

2) Within 6 months after the first stroke onset.

3) Aged between 40 and 70 years.

4) Stable vital signs, clear consciousness and good compliance to participate in the study.

5) Holden Walking Function Classification Grade 2 or above.

6) Sound cardiopulmonary function and tolerance to rehabilitation training.

7) Voluntarily sign the informed consent form.

②Exclusion Criteria

1) Orthopedic diseases, such as post-fracture and osteoarthritis.

2) Malignant progressive hypertension.

3) Malignant tumors.

4) History of thrombosis.

5) History of mental illness.

③Termination and Drop-out Criteria

1) The patient or their family voluntarily withdraws from the trial.

2) The researcher judges that continued participation may cause harm to the subject.

3) Severe complications or other diseases occur during the trial.

4) The patient has poor tolerance and cannot complete the study.

5) Failure to finish the study due to other reasons.

三、Research Methods：

①Subjects are inpatients with stroke admitted to our Department of Rehabilitation who meet the above criteria. Patients and their families will be fully informed of the study objectives, methods, potential benefits and risks. Those who sign the informed consent form will be enrolled.

Based on relevant literature, sample size is calculated via G*Power 3.1. With a medium effect size of 0.5, statistical power of 0.8 and significance level α of 0.05, the required sample size is 34 cases. Taking a 10%–15% drop-out rate into account, a total of 40 eligible subjects will be recruited. They will be divided into two groups (20 cases each) using the random number table method: the combined group receiving blood flow restriction training plus balance training, and the control group receiving balance training alone. There are no statistically significant differences between the two groups in terms of gender, age, stroke type, disease course, hemiplegic side and lower limb Brunnstrom stage ($P>0.05$).

②Training method：

1) Both groups receive symptomatic medication during hospitalization to control blood pressure, blood glucose and other indicators. Conventional rehabilitation therapy centered on neuromuscular facilitation techniques is also provided, including Bobath approach, proprioceptive neuromuscular facilitation (PNF) and motor relearning programme

2) On the basis of conventional rehabilitation therapy, the control group received

visual feedback balance training (Model: Prokin 252, Tecnobody, Italy). Patients could choose preferred balance training games, including table tennis, balance ball, skiing and fruit slicing. The training was conducted 20 minutes per day, 5 days a week, for a total of 4 weeks；

3) The experimental group received additional blood flow restriction training on the basis of the interventions for the control group. A pressurized blood flow restriction cuff (Theratools, Henan Heju Rehabilitation Technology Co., Ltd.) was applied to the proximal part of the affected thigh close to the trunk during balance training, with the pressure maintained at 160–200 mmHg. The inflation tube of the air pump was connected to the cuff valve for real-time pressure monitoring and adjustment.To guarantee safety, the patient's blood pressure, blood oxygen saturation and cuff pressure were monitored every 5 minutes throughout the training. Participants' subjective feelings such as numbness, pain and fatigue, as well as limb circumference and skin color changes were closely observed. The training duration was identical to that of the control group.

(3)**Rehabilitation Assessment Outcomes:**

All subjects underwent rehabilitation assessments twice, namely before treatment and after 4 weeks of intervention. The assessment indicators are as follows:

①**Primary outcomes：**

1) Balance assessment using balance testing and training system (Model Prokin 252, Tecnobody, Italy):Patients’ balance function and limits of stability were evaluated to assess fall risk by recording center-of-pressure parameters, including anteroposterior standard deviation, mediolateral standard deviation, mean anteroposterior movement velocity, mean mediolateral movement, sway path length and sway ellipse area. The limits of stability in standing position represent the range of dynamic balance; loss of balance and falls will occur once the body moves beyond this range. Directions with a smaller reachable range correspond to higher fall risk;

2)Gait analysis: The 3D gait analysis and training system (Model: Gait Watch) manufactured by Chongqing Hengshuo Medical Devices Co., Ltd. was adopted for evaluation. Patients’ walking ability was analyzed by examining cadence and joint

range of motion during walking:

②**Secondary outcomes：**

1)The lower-extremity subsection of the Fugl-Meyer Assessment (FMA-LE) was used to evaluate lower limb function in stroke patients；

2)Berg Balance Scale (BBS): to assess patients' balance function；

3)Timed Up and Go Test (TUGT): It is used to evaluate patients' functional walking speed; a shorter time indicates a faster walking speed.

4)Activities of daily living assessment: The Modified Barthel Index (MBI) was used to evaluate patients' activities of daily living.；

5)Blood test: D-dimer was detected to monitor the risk of thrombosis. Higher D-dimer concentration indicates a greater possibility of stable thrombosis. A D-dimer concentration less than 0.5 mg/mL can help rule out the risk of deep venous thrombosis.

6)Venous color Doppler ultrasonography: to examine whether thrombosis occurs in the affected lower limbs of patients.

(4) **Statistical analysis:** SPSS 26.0 software was used for statistical processing of all above assessment indicators.

(5) Flow chart of the study

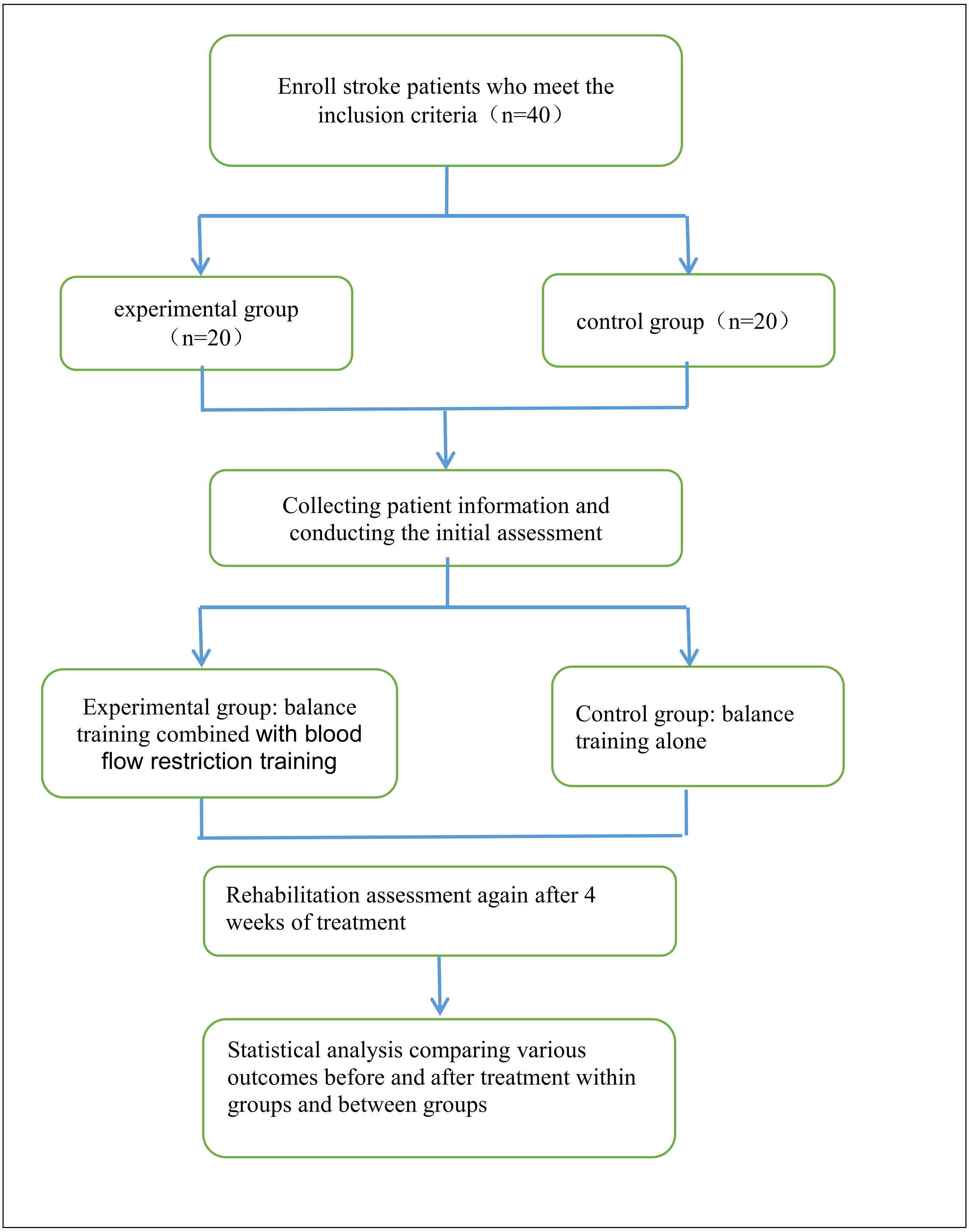
Enroll stroke patients who meet the inclusion criteria（n=40）
experimental group（n=20）
control group（n=20）
Collecting patient information and conducting the initial assessment
Experimental group: balance training combined with blood flow restriction training
Control group: balance training alone
Rehabilitation assessment again after 4 weeks of treatment
Statistical analysis comparing various outcomes before and after treatment within groups and between groups
